# Identifying and overcoming barriers to referral in advanced heart failure. A scientific statement of the Heart Failure Association (HFA) of the ESC

**DOI:** 10.1002/ejhf.70003

**Published:** 2025-08-17

**Authors:** Guillaume Baudry, Maria Generosa Crespo‐Leiro, Clément Delmas, Federica Guidetti, Marta Jimenez‐Blanco Bravo, Federica Valente, Maja Cikes, Nicolas Girerd, Finn Gustafsson, Gianluigi Savarese, Linda W. van Laake, Loreena Hill, Anne Kathrine Skibelund, Andreas Zuckermann, Marco Metra, Kevin Damman

**Affiliations:** ^1^ Université de Lorraine, INSERM, Centre d'Investigation Clinique Plurithématique 1433, Inserm U1116, CHRU de Nancy Nancy France; ^2^ INI‐CRCT (Cardiovascular and Renal Clinical Trialists) F‐CRIN Network Nancy France; ^3^ REICATRA, Université de Lorraine Vandoeuvre‐les‐Nancy France; ^4^ Cardiology Department Complexo Hospitalario Universitario A Coruña (CHUAC) A Coruña Spain; ^5^ Centro de Investigación Biomedica en Red Cardiovascular (CIBERCV) Madrid Spain; ^6^ Faculty of Medicine Universidade da Coruña (UDC) A Coruña Spain; ^7^ Intensive Cardiac Care Unit, Cardiology Department Rangueil University Hospital Toulouse France; ^8^ Department of Clinical Science and Education Södersjukhuset; Karolinska Institute Stockholm Sweden; ^9^ Department of Cardiology University Cardiovascular Center, Bern University Hospital, Inselspital Bern Switzerland; ^10^ Cardiology Department Hospital Universitario Ramón y Cajal, Centro de Investigación Biomedica en Red Cardiovascular (CIBERCV) Madrid Spain; ^11^ Department of Cardiology Erasme University Hospital, Université Libre de Bruxelles Brussels Belgium; ^12^ Department of Cardiovascular Diseases University of Zagreb School of Medicine & University Hospital Center Zagreb Zagreb Croatia; ^13^ Department of Cardiology Rigshospitalet, Copenhagen University Hospital Copenhagen Denmark; ^14^ Department of Cardiology University Medical Center Utrecht Utrecht The Netherlands; ^15^ School of Nursing and Paramedic Science Ulster University Londonderry UK; ^16^ ACNAP Science Committee member and ESC Patient Forum Representative Copenhagen Denmark; ^17^ Department of Cardiac and Thoracic Aortic Surgery Medical University of Vienna Vienna Austria; ^18^ Cardiology, ASST Spedali Civili, Department of Medical and Surgical Specialties, Radiological Sciences, and Public Health University of Brescia Brescia Italy; ^19^ University of Groningen, Department of Cardiology University Medical Centre Groningen Groningen The Netherlands

**Keywords:** Advanced heart failure, Referral, Mechanical circulatory support, Heart transplantation, Quality of care centres

## Abstract

The identification of patients with advanced heart failure (HF) remains challenging, often leading to delayed referrals and suboptimal use of advanced therapies such as long‐term mechanical circulatory support (MCS) or heart transplantation (HT). This delay contributes to worse outcomes and missed opportunities for timely intervention. Many eligible patients are not recognized early enough in their clinical trajectory, either due to the complexity of the condition, overlapping HF phenotypes, or limited awareness of referral criteria among non‐specialist clinicians. In this context, the aim of this scientific statement from the Heart Failure Association (HFA) of the ESC is to systematically identify and address the multifaceted barriers that hinder early recognition and referral for advanced HF care. These barriers span across different stakeholders—patients, caregivers, referring physicians, HF specialists, the academic community, and health authorities. The document proposes practical, stakeholder‐specific solutions to improve awareness, standardize referral criteria, integrate digital decision‐support tools, and structure care networks. Ultimately, the goal is to enable earlier access to specialized evaluation, ensure equitable use of HT and MCS when appropriate, and improve both survival and quality of life for patients living with advanced HF.

## Introduction

Heart failure (HF) affects over 15 million individuals across Europe and North America.^1^, ^2^, ^3^ Since the 2010s, a concerning rise in the incidence, and prevalence of HF has been observed, particularly among patients under 70 years of age who may require advanced therapies such as heart transplantation (HT) or long‐term mechanical circulatory support (MCS) including left ventricular assist devices (LVAD) or total artificial hearts (TAH).^2^, ^3^, ^4^, ^5^ This trend coincides with rising HF‐related mortality across all ages.^6^, ^7^ Advanced HF is the most severe stage of the disease, characterized by high mortality, debilitating symptoms and the need for such therapies.^8^, ^9^, ^10^ The estimated 1‐year survival rate is poor, with only about 50% of patients surviving, regardless of HF phenotype.^11^ Although data remain scarce in this specific population, the global prevalence of advanced HF is estimated to range from 0.2% to 14%, depending on the definition and population studied.^8^, ^12^, ^13^

Heart transplantation is recommended for eligible patients with advanced HF, and MCS should be considered to reduce mortality risk and improve symptoms, according to the 2021 European Society of Cardiology (ESC) guidelines for the diagnosis and treatment of acute and chronic HF.^9^ Despite these recommendations and improvement of MCS technologies, use of HF advanced therapies has globally stagnated and remains insufficient to meet patient needs.^14^, ^15^, ^16^ Indeed, the number of HT and MCS device implants per capita varies significantly between countries, with heterogeneous trends over time.^14^

One contributing factor to this gap is the challenge of accurately identifying appropriate candidates and ensuring their timely referral to specialized centres for comprehensive evaluation. Effective management of advanced HF depends on early recognition of patients and prompt referral to advanced centres, where the expertise and logistical support for HT and MCS are centralized, allowing resources and skills to be pooled.^8^, ^17^, ^18^ Early identification is also crucial to initiate timely discussions around palliative care and advance care planning, ensuring that patients and families are adequately supported and that care aligns with their goals and preferences before critical deterioration occurs.^19^, ^20^

In addition to their role in delivering surgical therapies, advanced HF centres also serve as hubs for research and innovation, supporting the development and optimization of care strategies beyond transplantation and mechanical support. These centres, although not exclusive in this function, are also key environments for delivering holistic, patient‐centred care, including the timely integration of palliative support when appropriate.

The aim of this scientific statement of the Heart Failure Association (HFA) of the ESC is to identify and address the barriers involving patients, referring clinicians, advanced HF teams, the academic community, and health authorities that hinder the timely recognition of advanced HF and appropriate referral to specialized centres. By proposing targeted, stakeholder‐specific solutions, this initiative seeks to facilitate equitable access to advanced HF therapies, streamline referral pathways, and ultimately reduce HF‐related mortality in eligible patients (*Graphical Abstract*).

## Addressing academic and research gaps in advanced heart failure

### Indicators and definition of advanced heart failure and overlap with other heart failure presentations

Advanced HF is a heterogeneous clinical condition that requires both early recognition and appropriate referral. To support this process, several practical severity indicators have been proposed (*Table* *1*) to help clinicians working within all non‐transplant settings (i.e. general practitioners, specialist nurses, cardiologists) to identify patients who may benefit from assessment in an advanced HF centre.^8^, ^21^, ^22^, ^23^, ^24^ Many of these indicators are summarized in the mnemonic ‘I NEED HELP’ (*Table* *2*), which serves as a practical tool to prompt timely referral discussions.^25^

**Table 1:** Clinical indicators of advanced heart failure that should trigger a referral to advanced heart failure centres proposed by different international societies documents

Markers of advanced HF	Heart Failure Association of the ESC (2018)^8^	American Heart Association/ American College of Cardiology (2022)^21^	Heart Failure Society of America (2015)^22^
Inotropes	Prior inotrope use	Need for intravenous inotropic therapy	Need for intravenous inotropic therapy
Functional capacity	Inability to perform CPT	Peak VO_2_, <14 ml/kg/min or <50% predicted, 6MWD <300 m Inability to walk 1 block on level ground	Peak VO_2_ <14 ml/kg/min or <50% of predicted, 6MWD <300 m Inability to perform ADL
NYHA class	NYHA class III–IV	Persistent NYHA class III–IV	Progressive/persistent NYHA class III–IV
NT‐proBNP	NT‐proBNP >1000 pg/ml	–	–
HF hospitalizations	>1 HHF in last 12 months	Repeated HHF or emergency department visits for HF in the past 12 months	≥ 2 HHF within 12 months >2 unscheduled visits or access to ED within 12 months
Ventricular arrhythmias	Ventricular arrhythmias/ICD shocks	Refractory or recurrent ventricular arrhythmias, frequent ICD shocks	Recurrent refractory ventricular tachyarrhythmias, frequent ICD shocks
Right HF	–	Worsening right HF or secondary pulmonary hypertension	Worsening right HF and secondary pulmonary hypertension
Diuretics and congestion	Increasing diuretic requirement	Recent need to escalate diuretics to maintain volume status, often reaching daily furosemide equivalent dose >160 mg/day or use of supplemental metolazone therapy Refractory clinical congestion	Diuretic refractoriness associated with WRF
Intolerance to GDMT	Intolerance to optimal dose of any GDMT	Intolerance to RAASi because of hypotension or WRF Intolerance to BB because of worsening HF or hypotension	Circulatory‐renal limitation to RAASi or BB
CRT non‐responder	Clinically CRT non‐responder	–	–
Renal and liver dysfunction	eGFR <45 ml/min SCr ≥160 μmol/L K >5.2 mmol/L or <3.5 mmol/L Abnormal liver function Low albumin	Progressive deterioration in renal or hepatic function	Progressive renal or hepatic end‐organ dysfunction
Scores mortality risk	MAGGIC predicted survival ≤80% at 1 year^23^ SHFM predicted survival ≤80% at 1 year^24^	Increased predicted 1‐year mortality (e.g. >20%) according to HF survival models (e.g. MAGGIC,^23^ SHFM^24^)	Increased 1‐year mortality (e.g. 20–25%) predicted by HF survival models (e.g. SHFM,^24^ HFSS, etc.)
Cachexia	Cachexia Unintentional weight loss	Cardiac cachexia	Cardiac cachexia
Hyponatraemia	Hyponatraemia	Persistent hyponatraemia (serum sodium <134 mEq/L)	Persistent hyponatraemia (serum sodium <134 mEq/L)
Hypotension	SBP ≤90 mmHg	Frequent SBP <90 mmHg	–
Echo parameters	EF ≤30% Large area of akinesis/dyskinesis or aneurysm Moderate‐severe mitral regurgitation RV dysfunction PAP ≥50 mmHg Moderate‐severe tricuspid regurgitation Difficult to grade aortic stenosis IVC dilated or without respiratory variation	–	–

6MWD, 6‐min walk distance; ADL, activities of daily living; BB, beta‐blocker; CPT, cardiopulmonary test; CRT, cardiac resynchronization therapy; ED, emergency department; EF, ejection fraction; eGFR, estimated glomerular filtration rate; ESC, European Society of Cardiology; GDMT, guideline‐directed medical therapy; HF, heart failure; HHF, hospitalization for heart failure; HFSS, Heart Failure Survival Score; ICD, implantable cardioverter‐defibrillator; IVC, inferior vena cava; MAGGIC, Meta‐Analysis Global Group in Chronic Heart Failure; NT‐proBNP, N‐terminal pro‐B‐type natriuretic peptide; NYHA, New York Heart Association; PAP, pulmonary artery pressure; RAASi, renin–angiotensin–aldosterone system inhibitor; RV, right ventricular; SBP, systolic blood pressure; SCr, serum creatinine; SHFM, Seattle Heart Failure Model; VO_2_, oxygen uptake; WRF, worsening renal function.

**Table 2:** ‘I NEED HELP’ criteria

I	**I**notropes	Previous or ongoing requirement for inotropes
N	**N**YHA class/**N**Ps	Persisting NYHA class III–IV/persistently high NPs
E	**E**nd‐organ dysfunction	Worsening liver or renal dysfunction in the setting of HF
E	**E**F	EF <20%
D	**D**efibrillator shocks	Recurrent appropriate defibrillator shocks
H	**H**F hospitalization	>1 HF hospitalization within 12 months
E	**E**dema/**E**scalating diuretics	Persisting fluid overload and/or increasing diuretic requirement
L	**L**ow blood pressure	Consistently SBP <100 mmHg
P	**P**rognostic medication	Inability to up‐titrate (or need to decrease/cease) GDMT

EF, ejection fraction; GDMT, guideline‐directed medical therapy; HF, heart failure; NYHA, New York Heart Association; NP, natriuretic peptide; NT‐proBNP, N‐terminal pro‐B‐type natriuretic peptide; SBP, systolic blood pressure.

Complementary to these screening indicators, a more stringent definition is needed to guide the appropriate use of advanced therapies such as HT or long‐term MCS. Various international societies have proposed definitions of advanced HF, consistently recognizing it as the most severe stage of the disease, marked by persistent symptoms despite guideline‐directed medical therapy (GDMT) (*Table* *3*).^8^, ^21^, ^22^, ^26^ The definition of advanced HF outlined in the 2018 HFA‐ESC position statement, is now referenced in both European and American HF guidelines, specifies that patients must meet four simultaneous criteria: New York Heart Association (NYHA) class III–IV, severe cardiac dysfunction, at least two episodes of acute HF, low output or ventricular arrhythmias within 12 months, and severely impaired functional capacity (*Table* *3*).^8^, ^9^, ^21^

**Table 3:** Comparison of most recent advanced heart failure and stage D heart failure definitions across different international societies

Heart Failure Association of the ESC (2018)^8^	American Heart Association/ American College of Cardiology (2022)^21^	Heart Failure Society of America (2015)^22^
All the 4 following criteria must be present despite GDMT: **Severe** and persistent HF **symptoms** (NYHA class III advanced‐IV) Severe cardiac dysfunction: EF ≤ 30% or Isolated RV failure or Non‐operable severe valve abnormalities or congenital abnormalities or Persistently high/increasing NPs + severe diastolic dysfunction 3 >1 unplanned visit or hospitalization in the last 12 months due to: **Episodes of pulmonary or systemic congestion** requiring high‐dose intravenous diuretics or diuretic combinations or **Episodes of low output** requiring inotropes or vasoactive drugs or **Malignant arrhythmias** causing >1 unplanned visit or hospitalization in the last 12 months 4 **Severe impairment of exercise capacity** estimated to be of cardiac origin with: Inability to exercise or 6MWD <300 m or Peak VO_2_ <12–14 ml/kg/min	Marked HF **symptoms** that interfere with daily life and with **recurrent hospitalizations** despite attempts to optimize GDMT.	Presence of progressive and/or persistent **severe signs and symptoms of HF** despite GDMT. It is generally accompanied by **frequent hospitalization**, severely **limited exertional tolerance**, and poor quality of life and is associated with high morbidity and mortality. The progressive decline should be primarily driven by HF.

6MWD, 6‐min walk distance; EF, ejection fraction; GDMT, guideline‐directed medical therapy; HF, heart failure; NYHA, New York Heart Association; NP, natriuretic peptide; RV, right ventricular; VO_2_, oxygen uptake.

The strength of this definition lies in its clarity, feasibility, and emphasis on symptom severity rather than cardiac function alone. In contrast with earlier definitions, it recognizes that advanced HF can occur across all ejection fraction (EF) profiles, including HF with reduced, mildly reduced or preserved EF, isolated right ventricular dysfunction, or non‐operable congenital or valvular diseases.^8^, ^27^ Despite its simplicity, a recent survey of almost 500 cardiologists, (including HF cardiologists, general cardiologists and other participants) revealed that less than 50% use these criteria regularly in their daily clinical practice.^28^

Since neither the HFA‐ESC definition nor the mnemonic ‘I NEED HELP’ had been formally validated, Pagnesi *et al*.^11^ recently evaluated their prognostic impact in the HELP‐HF registry, a retrospective study conducted at four high‐volume centres in Italy. The study enrolled 1149 in‐ and outpatients who met at least one ‘I NEED HELP’ criterion. A higher cumulative number of ‘I NEED HELP’ criteria was independently associated with a higher risk of the primary endpoint (all‐cause death or HF hospitalization).^29^ Most of these criteria, when taken individually, were associated with the outcomes. The 1‐year rates of all‐cause mortality increased from 20% in patients with two ‘I NEED HELP’ criteria to 60% in those with six.

Among this preselected cohort, 16.8% met the full HFA‐ESC definition and these were at significantly higher risk of HF hospitalization or mortality compared to those who did not meet all four HFA‐ESC criteria, even after multivariable adjustments. The 1‐year rates of the primary composite endpoint, as well as its components, all‐cause mortality alone and HF hospitalization alone, were 69.3%, 46.5%, and 39.5%, respectively in patients meeting the full HFA‐ESC definition versus 41.8%, 21.5%, and 26.9% in those not meeting the definition.

Taken together, this real‐world evidence suggests a complementary role of both approaches. The ‘I NEED HELP’ criteria are effective for early identification and risk stratification of patients who may require further assessment, while the HFA‐ESC definition identifies those at the highest risk who are most likely to benefit from advanced therapies. Using both tools in parallel may optimize the timely referral and management of advanced HF patients. While this approach shows promise, further validation is required, particularly within a population age‐eligible for HT or MCS. The study also highlighted a limitation of the HFA‐ESC definition: since it was primarily designed to identify chronic advanced HF, it may fail to capture de novo or acute presentations requiring urgent intervention. A substantial proportion of patients with INTERMACS profiles 1–3 did not meet all four criteria.^29^

Each hospitalization for acute HF carries a mortality risk of 5–7% and is often followed by clinical deterioration within 6 months, increasing the risk of readmission and death.^30^, ^31^, ^32^ Worsening HF can also lead to progressive decline without hospitalization, resulting in renal dysfunction or cachexia and compromising the patient's eligibility for HT or MCS.^32^ Early identification of these scenarios is therefore critical.

In addition, the HFA‐ESC definition requires at least two unplanned hospitalizations, which may not apply to patients who, from the first episode, cannot be discharged home or survive without immediate HT or durable MCS. Delayed referral in such cases jeopardizes patient safety. Recent studies also show almost similar prognoses between worsening HF and advanced HF patients, and distinguishing between these profiles in practice is often difficult.^33^ This underscores the importance of early referral based on clinical trajectory rather than strict criteria. Indeed, urgent interventions are typically associated with worse outcomes, further reinforcing the need for timely referral and planned evaluation. Moreover, identifying a suitable graft for HT requires time, even in high‐priority cases, and preoperative assessment and optimization require adequate preparation to improve post‐transplant outcomes.

The definition may lack sensitivity for certain phenotypes, or for instance, it does not account for patients with low cardiac output who may present with severely reduced peak oxygen consumption despite the absence of recent hospitalizations, potentially due to low pulmonary pressures. Some authors describe this advanced phase of HF as being governed by the ‘law of Laplace’, where increased wall stress—rather than neurohormonal activation—drives disease progression.^34^ In this context, GDMT often becomes ineffective due to intolerance, requiring interventions aimed at reducing wall stress (e.g. LVAD) or ultimately replacing the heart through HT or a TAH.^34^ Without such therapies, the disease frequently progresses rapidly toward death. Moreover, progressive genetic cardiomyopathies (e.g. BAG3, LMNA, FLNC), which are associated with poor prognosis and may require earlier consideration of advanced therapies, are also not captured by the current definition.^35^

**Figure 1:**
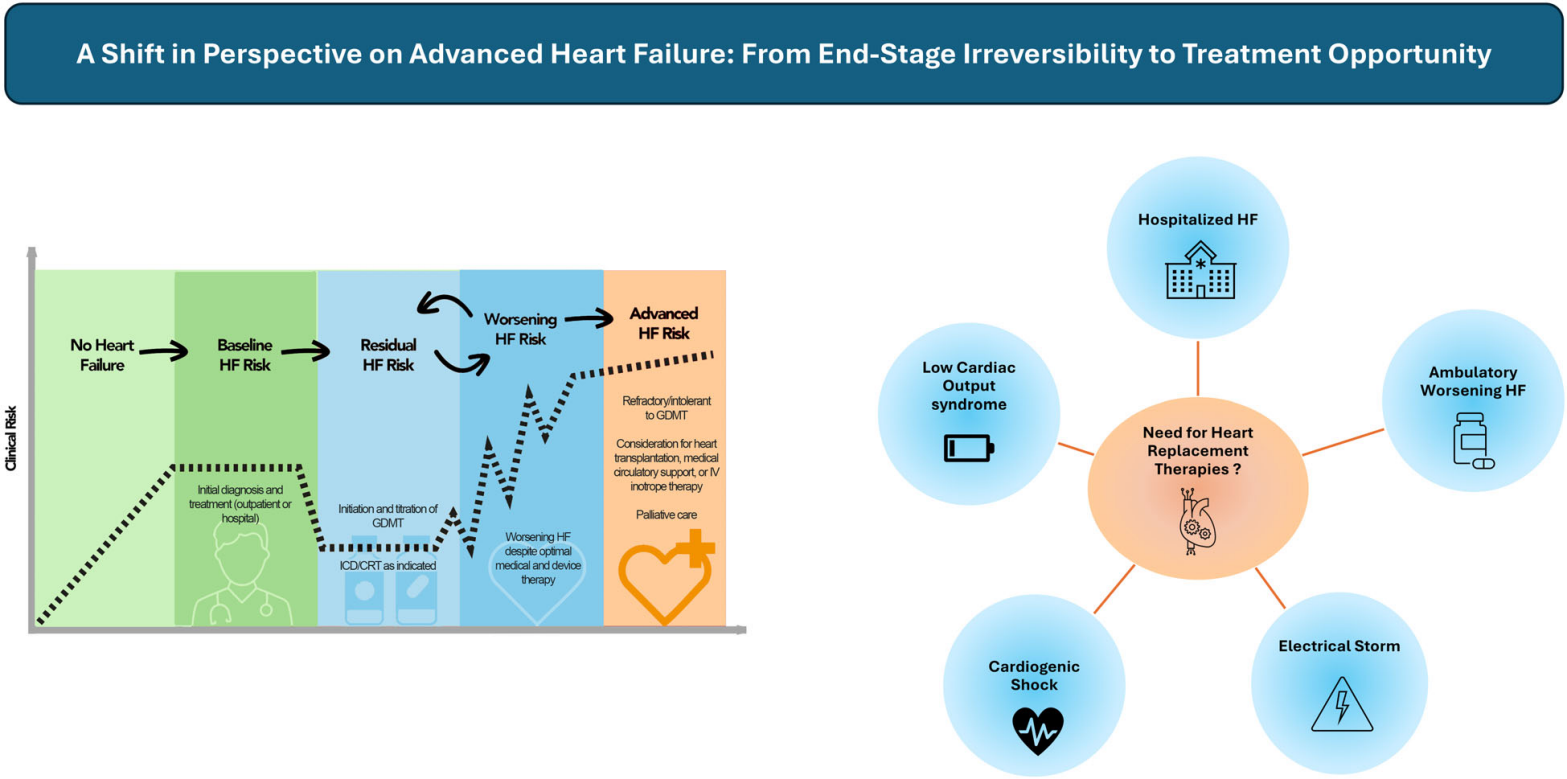
From the longitudinal ‘end‐stage’ concept to the ‘transplant or long‐term device‐required’ paradigm: a redefinition of advanced heart failure. CRT, cardiac resynchronization therapy; GDMT, guideline‐directed medical therapy; HF, heart failure; ICD, implantable cardioverter‐defibrillator; IV, intravenous.

*Figure* *1* illustrates this paradigm shift from a terminal‐stage view to a more functional, intervention‐based understanding of advanced HF, focused on identifying patients in need of durable mechanical support or transplantation regardless of EF or chronicity.

**Figure 2:**
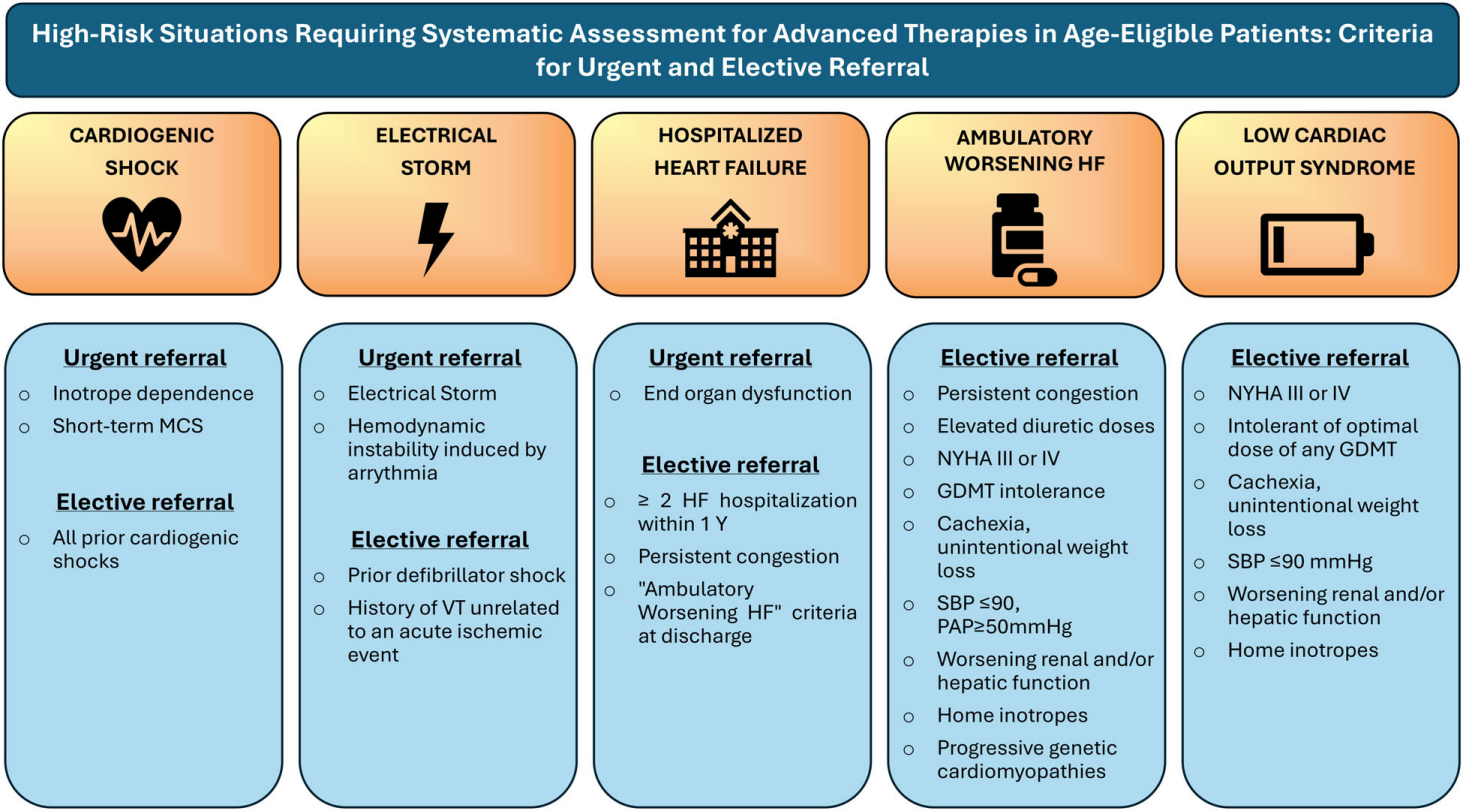
High‐risk situations requiring systematic assessment for advanced therapies in age‐eligible patients: criteria for urgent and elective referral. GDMT, guideline‐directed medical therapy; HF, heart failure; MCS, mechanical circulatory support; NYHA, New York Heart Association; PAP, pulmonary artery pressure; SBP, systolic blood pressure; VT, ventricular tachycardia; Y, year.

In conclusion, the current HFA‐ESC definition remains a valuable tool for identifying chronic patients at very high risk and guiding therapeutic decisions. However, waiting until all four severity criteria are met may delay referral and compromise outcomes. Moreover, the definition lacks sensitivity in unstable or de novo cases. Transitioning toward simplified referral tools and a definition that emphasizes early recognition of clinical scenarios requiring advanced therapies is essential to improve both timely referral and appropriate candidate selection (*Figure* *2*).

### Absence of randomized trial evidence to guide referral or advanced therapies

The management of HF has been shaped by over 40 years of randomized controlled trials (RCTs), which have defined target populations and expected benefits. However, in the context of advanced HF, there is a notable lack of recent RCTs to guide the optimal timing of referral to advanced centres or to identify the most suitable candidates for HT or MCS, especially considering the profound therapeutic advancements in device technology over recent years. The 2021 ESC guidelines for the diagnosis and treatment of acute and chronic HF explicitly highlighted the need for randomized evidence in this population.^9^

Centrifugal‐flow LVADs have shown superior 5‐year survival rates (~60% vs. ~45%) compared to axial‐flow pumps, along with improved haemocompatibility, fewer device‐related complications, and enhanced quality of life (QoL). These findings, supported by both RCTs and real‐world registries, have encouraged earlier implantation of LVADs, particularly in high‐risk ambulatory patients with advanced HF.^16^, ^36^, ^37^ To address existing gaps in evidence, three ongoing European trials are comparing GDMT with early long‐term LVAD implantation: SweVAD (NCT02592499) for destination therapy, Early‐VAD (NCT02387112) as a bridge to transplant, and Ambu‐VAD (NCT04768322) evaluating both strategies.

In addition, the global TEAM‐HF trial (NCT06526195) has been launched to compare the HeartMate 3® LVAD with GDMT in ambulatory NYHA class IIIB/IV HF patients identified as high‐risk based on pulmonary pressure monitoring. These studies aim not only to assess the benefits of early LVAD implantation but also to refine referral strategies and timing by improving patient selection criteria for long‐term MCS or transplantation in advanced ambulatory HF.

### Guideline familiarity

The reasons for referral delays remain unclear but may involve limited awareness of referral criteria. To address these challenges, the 2021 ESC guidelines for the diagnosis and treatment of acute and chronic HF introduced a dedicated section on advanced HF, covering its epidemiology, diagnosis, prognosis, and treatment.^9^ However, despite significant efforts by scientific societies, there remains considerable room for improving guideline awareness among cardiologists.^14^, ^28^

According to a recently published survey, approximately half of non‐HF specialists had a limited knowledge of HF guidelines and only 44% ‘regularly or always’ used HFA‐ESC criteria to recognize advanced HF patients, which underscores the limited adoption of these guidelines within the broader cardiology community. It is of note that only half of the cardiologists in this survey were aware of the recommendation levels for advanced HF therapies, including HT and MCS.^28^

General cardiologists or HF specialists have a key role in the diagnosis and treatment of HF patients.

Some proposals to improve the implementation of HF guidelines and facilitate timely referrals to advanced therapy centres include:

- Annual meeting between hub & spoke centres: regular meetings help update scientific knowledge and foster collaboration while sharing outcomes enhances mutual learning and accountability.
- Incorporate guidelines into clinical practice tools: integrate HF guidelines into electronic health record systems and clinical decision‐support tools to provide real‐time guidance during patient care.
- Educational workshops and seminars: interactive workshops led by experts foster engagement and offer opportunities for real‐time discussions, enhancing understanding and practical application.
- Continuing medical education (CME) programs: accredited CME programs ensure up‐to‐date knowledge while motivating clinicians to stay current with guideline updates.
- Online training modules: flexible, interactive modules provide accessible learning opportunities for busy professionals and help reinforce key concepts.

Expanding awareness of advanced HF guidelines within the cardiology community is crucial to bridging care gaps, optimizing therapies, and ensuring timely referrals, ultimately improving patient outcomes.

## Challenges in network structuring and access to improve advanced heart failure care

In a recent survey of nearly 500 cardiologists, 16.5% reported having no dedicated team to discuss long‐term MCS or HT. Furthermore, 18.5% and 19.6% found it difficult or very difficult to discuss or refer patients to advanced HF centres, underscoring the importance of network structuring to streamline referrals for the most severe cases.^28^ Similarly, despite its importance in facilitating timely referrals, access to cardiopulmonary exercise testing (CPET), which provides critical insights into cardiovascular reserve and prognosis, remains inconsistent, particularly among general practitioners and cardiologists.^8^, ^38^ Surveys indicate that up to half of general cardiologists face challenges in accessing CPET or referring patients to advanced HF units, contributing to variability in care and delays in treatment.^28^ Expanding CPET access is essential and could be achieved by decentralizing access to CPET at regional centres through staff training or by establishing satellite testing facilities connected to central hubs. Alternatively, mobile CPET, which shows strong correlation with standard CPET parameters, can service multiple spoke centres, expanding access to this critical evaluation tool.^39^

Establishing specialized HF networks is crucial to ensuring that patients have equitable access to advanced HF centres, regardless of their place of residence. European and North American guidelines advocate a three‐tiered hub‐and‐spoke model of healthcare providers—comprising advanced‐level centres, specialized HF centres, and community‐based centres—to address the full spectrum of HF severity and patient needs.^8^, ^9^, ^21^

Although the multi‐level care model is broadly endorsed, there is ongoing debate regarding the optimal design and implementation of HF network services. A well‐structured HF network should enable optimal and consistent patient management across the network, with centre selection tailored individually based solely on the severity of the patient's condition. Achieving this vision requires streamlined and efficient communication between all levels of care centres.

In pursuit of this goal, the HFA‐ESC and the National HF Societies have developed ICARe‐HF (Improving Care through Accreditation and Recognition in HF).^40^ This accreditation program is designed to enhance outcomes for HF patients across Europe and beyond by evaluating the performance of individual centres, institutions, and clinics according to established standards. These standards are aligned with the HFA‐ESC curriculum and ESC guidelines.^9^, ^40^, ^41^

**Figure 3:**
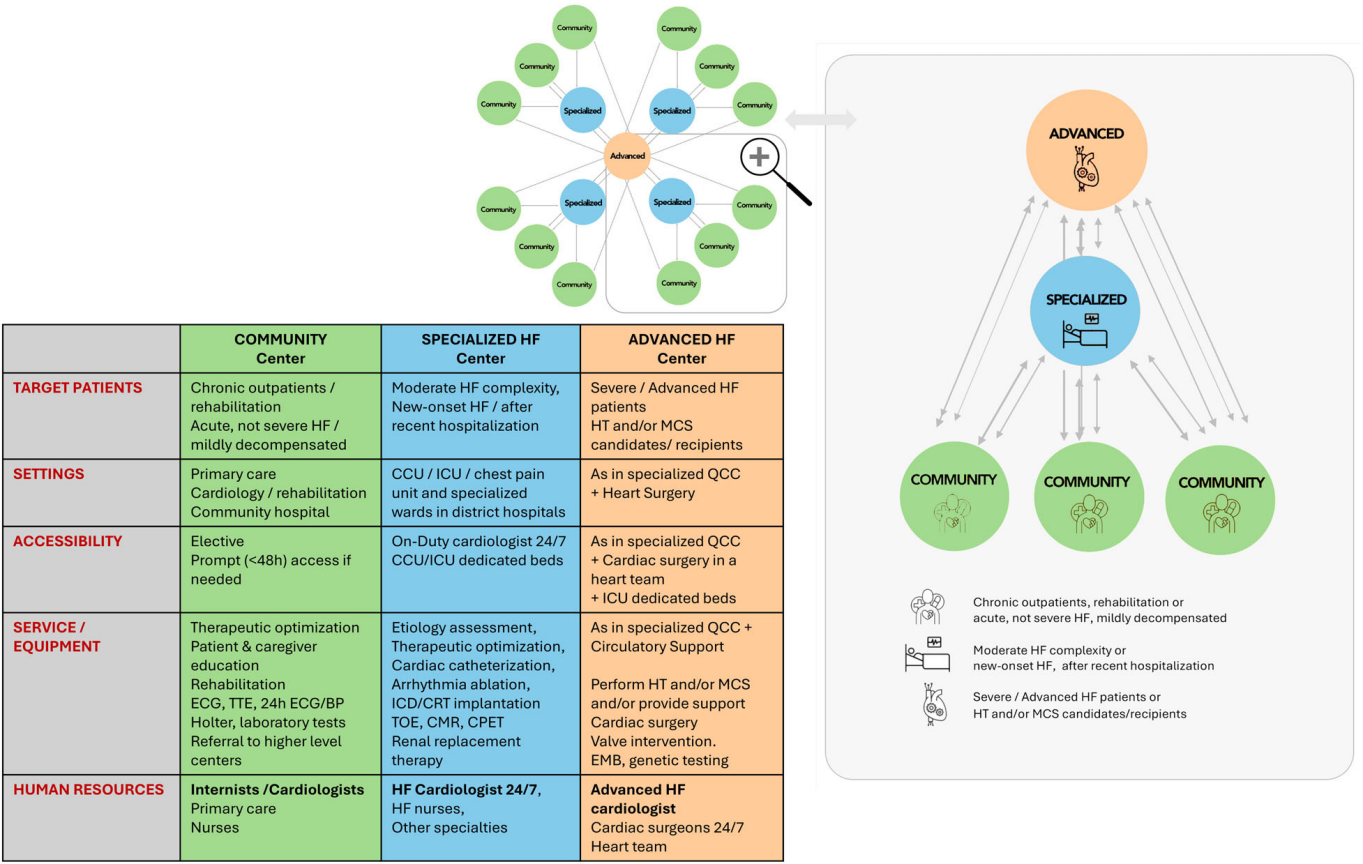
Hub‐and‐spoke network organization for heart failure care: distribution of patients across community, specialized, and advanced centres based on severity. BP, blood pressure; CCU, coronary care unit; CMR, cardiac magnetic resonance; CPET, cardiopulmonary exercise testing; CRT, cardiac resynchronization therapy; ECG, electrocardiogram; EMB, endomyocardial biopsy; HF, heart failure; HT, heart transplantation; ICD, implantable cardioverter‐defibrillator; ICU, intensive care unit; MCS, mechanical circulatory support; QCC, quality of care centre; TOE, transoesophageal echocardiography; TTE, transthoracic echocardiography.

The three categories of HFA Quality of Care Centres proposed are (*Figure* *3*):

- Community Quality of Care Centres: these centres encompass primary care facilities, local cardiologists, community hospitals, and rehabilitation centres. Their role is to conduct the initial assessments and manage HF patients within the community, focusing on the optimization of therapy for those with chronic HF.
- Specialized Quality of Care Centres: these centres receive referrals from Community Quality of Care Centres and typically include district hospitals equipped with intensive care units and cardiac catheterization labs. Although the presence of electrophysiology and device implantation capabilities is highly recommended, it is not mandatory. Specialized centres provide advanced diagnostic assessments and treatments and manage acute or decompensated HF patients of intermediate complexity.
- Advanced Quality of Care Centres: serving as national reference facilities, these centres deliver inpatient care for patients with advanced or severely decompensated HF, including those eligible for HT or MCS.

By establishing a network of accredited centres, the ICARe‐HF program aims to foster consistency in HF care across diverse institutions, a critical factor for improving patient outcomes on a broad scale.

Each country should develop standardized communication pathways and organizational frameworks for advanced HF centres to ensure that referrals are tailored to each patient's unique characteristics and clinical needs.^8^, ^42^ While some aspects of these networks are well‐defined, such as the transfer of patients with cardiogenic shock, where a dedicated shock team and pre‐established communication channels have been shown to reduce 30‐day mortality significantly, other areas require further structuring.^42^, ^43^ For instance, referral processes for patients with new‐onset HF, complications, or worsening symptoms during ambulatory care are often ambiguous, resulting in delays in accessing advanced HF services.

Telemedicine can play a pivotal role by facilitating real‐time consultations between community, specialized, and advanced centres, enabling both immediate assessment of patients who may require advanced therapies and remote monitoring of those living far from advanced centres.^44^

Integrating multidisciplinary teams and enhancing communication between hub‐and‐spoke centres are essential for optimizing advanced HF care (*Figure* *3*).^8^, ^40^ Shared‐care models involving both community and advanced centre teams align care with current guidelines and evidence. Regular multidisciplinary meetings enhance collaborative decision‐making, which is vital for tailoring treatment strategies.

These centres play a pivotal role in guiding treatment strategies, ensuring that decisions are made collaboratively with the patient and their referring cardiology team, whether from a community practice or a specialized centre. By centering the patient in the decision‐making process, advanced HF centres provide more tailored, informed, and holistic care for individuals facing complex HF conditions.^17^

## Improving referral pathways for clinicians in advanced heart failure care

Most clinicians and referral teams are not specifically dedicated to advanced HF and typically manage a broad spectrum of cardiovascular conditions. As a result, maintaining focused expertise on this complex and infrequently encountered syndrome can be challenging. Informed decision‐making regarding HT or long‐term MCS requires not only a clear understanding of the long‐term outcomes, benefits, and potential complications of these therapies, but also an accurate, real‐time assessment of the patient's individual risk associated with continuing medical therapy alone.^17^, ^45^, ^46^ This evaluation is further complicated by the availability of complementary advanced options in expert centres, including percutaneous valve interventions, invasive pulmonary pressure monitoring, and structured remote follow‐up strategies, among others. Although 1‐year survival rates for HT and LVAD now approach 90%, identifying patients who are most likely to benefit remains a significant challenge for clinicians.

While several prognostic scores (such as Seattle HF score, MECKI, MAGGIC, or BCN Bio‐HF) are available, they are rarely used.^47^ These tools were not specifically designed for younger, device‐eligible patients, may rely on outdated data, and often require inputs that are not readily available during outpatient visits. Even when used, they lack clear thresholds to guide referral decisions to advanced centres.

Clinicians play a central role in guiding patients toward advanced HF care. Their ability to inform, reassure, and communicate confidence in the benefits of specialized therapies is key to overcoming patient hesitation and ensuring timely referral to expert centres. To fulfill this role, they must be familiar with the clinical outcomes and trust the value of these advanced treatments. However, a recent study revealed substantial variability in clinicians' perceptions of these therapies. While most cardiologists—regardless of subspecialty—were able to recognize a textbook case of advanced HF and accurately assess the risks of hospitalization and death, their attitudes toward MCS differed significantly. For example, 30% of general cardiologists, compared to only 10% of HF specialists, considered LVAD implantation premature or inappropriate in the same clinical scenario. These differences likely reflect disparities in familiarity with long‐term outcomes: only 22% of general cardiologists were aware of the 5‐year survival rate (58%) associated with centrifugal‐flow LVADs, compared to 42% of HF specialists. Awareness of bleeding and stroke risks related to MCS was also limited in both groups.^28^ Clinicians' personal beliefs and perceptions of treatment risks and benefits can directly influence their therapeutic recommendations and referral decisions. In the same survey, one‐third of cardiologists viewed the benefits of long‐term MCS as limited due to its complications. Notably, HF specialists expressed greater willingness to consider MCS for themselves or a family member, suggesting that personal trust in the therapy shapes both professional recommendations and patient counselling.^28^

To overcome these challenges, several approaches can be implemented. Structured screening based on simple, objective criteria can improve clinician awareness and facilitate the timely identification of patients eligible for HT or long‐term MCS. The SEE‐HF study demonstrated that among patients with cardiac resynchronization therapy and/or an implantable cardioverter‐defibrillator, structured screening led to the identification of advanced HF in routine settings, with 26% of those undergoing detailed evaluation found eligible for HT or LVAD—highlighting a significant under‐recognition of candidates for advanced therapies.^18^ The ongoing SAINTS study (Screening for Advanced heart failure IN stable ouTpatientS, NCT05299879) aims to assess the prevalence of advanced HF in symptomatic outpatients with HF and reduced EF using standardized screening protocols.

To support broader implementation, such screening could be embedded into electronic medical records using automated alerts or decision‐support tools that flag patients based on predefined parameters (e.g. persistent NYHA class III–IV, repeated hospitalizations, or low left ventricular EF despite optimal GDMT). Integrating simple screening checklists into follow‐up workflows or device interrogations could also help standardize identification across centres and reduce variability in referral practices.

In parallel, educational efforts are needed to improve clinician familiarity with the long‐term outcomes of HT and MCS. A better understanding of survival rates, QoL improvements, and complication profiles is essential to build trust in these therapies and support informed, confident referral decisions.

## Engaging patients and caregivers in the advanced heart failure care pathway

Despite clear benefits on QoL and clinical outcomes, advanced therapies come with specific risks, substantial lifestyle changes, and increased caregiving needs. HT requires lifelong immunosuppression, with potential complications including infections, nephrotoxicity, and cancer.^48^ MCS involves lifelong anticoagulation, with associated risks of bleeding and stroke, and dependence on external components such as batteries and percutaneous cables, which carry an ongoing risk of infection. Patients must also be capable of managing the device daily, including responding to alarms and caring for the driveline.^49^ These constraints often necessitate major adjustments in personal, social, professional, and sexual life, and can reduce autonomy while increasing reliance on caregivers.^50^

A key aspect of shared decision‐making in advanced HF is ensuring that patients fully understand the evolving balance of benefits and risks associated with these therapies.^51^, ^52^ This becomes particularly complex in cases of abrupt disease onset, such as cardiogenic shock following myocardial infarction, where patients have not experienced prior QoL deterioration. In such cases, the potential QoL gains from HT or MCS may be less immediately apparent compared to their baseline health status.

In advanced HF, patients frequently underestimate the severity of their condition, leading to significant discrepancies with physician assessments and potentially delaying appropriate referral and decision‐making. In the MedaMACS (Medical Arm for Mechanically Assisted Circulatory Support) cohort, which included 161 patients, physicians classified 69% of patients as high‐risk, while only 14% of patients perceived themselves as such. Interestingly, there were no statistically significant differences in survival rates or the need for surgical HF therapies between cohorts stratified by patient‐perceived high versus low risk. Additionally, risk perception did not correlate with rehospitalization frequency.^53^ In a cluster multicentre randomized trial conducted in the United States involving 407 ambulatory advanced HF patients, 73.7% exhibited discordance between patient and physician prognostic estimates. Specifically, patients often reported a prognosis of survival greater than 1 year when their physician estimated survival at less than 1 year. This subgroup of patients tended to report a lower symptom burden in terms of both number and severity. Notably, only 4.9% of patients with an estimated survival of less than 1 year were aware of and agreed with their clinician's assessment.^54^ In the REVIVAL (Registry Evaluation of Vital Information for Ventricular Assist Devices in Ambulatory Life) study, which included 400 ambulatory advanced HF patients, participants self‐estimated their remaining life expectancy at 7 years. Baseline QoL was associated with INTERMACS profile but only modestly correlated with patients' self‐assessed life expectancy (correlation coefficients ranging from 0.35 to 0.45). Furthermore, patient preferences for LVAD therapy were independent of age, QoL score, and self‐assessed life expectancy.^55^

Given the complexity of advanced HF therapies and the cognitive, emotional, and practical challenges they entail, caregivers play a critical yet often under‐recognized role throughout the continuum of care.^56^, ^57^, ^58^ They serve as essential partners in daily disease management, assisting with symptom monitoring, equipment handling, and emergency response, while also supporting patients in understanding their condition and navigating clinical decisions.^56^, ^58^ Their involvement is particularly important when patients underestimate the severity of their illness or struggle to process complex medical information. Before, during, and after interventions such as HT or MCS, caregivers help patients comprehend treatment options, weigh risks and benefits, and maintain adherence to therapy. In the pre‐referral phase, they are often key in identifying clinical decline, encouraging timely specialist consultation, and motivating patients to accept referral to tertiary care centres. By bridging the gap between physician assessments and patient perceptions, caregivers facilitate more accurate risk communication, reduce delays in referral, and contribute to more informed and patient‐centred decision‐making.^56^, ^58^

Patients with more advanced HF—reflected by lower INTERMACS profiles—were significantly more likely to express interest in receiving an LVAD, even after adjusting for age, HF aetiology, and prior ventricular assist device knowledge. A high burden of prior hospitalizations also independently increased the likelihood of patients stating they would ‘definitely want’ a device, whereas factors such as HF duration, prior cardiac surgery, or QoL scores were not significantly associated with LVAD preference.^59^

To support informed decision‐making, risk communication should include side‐by‐side comparisons of treatment options (e.g. HT vs. LVAD) and non‐intervention strategies (e.g. LVAD vs. palliative care), as recommended by the International Patient Decision Aid Standards.^60^ These comparisons must combine both general and personalized risk assessments to be meaningful and actionable.

**Figure 4:**
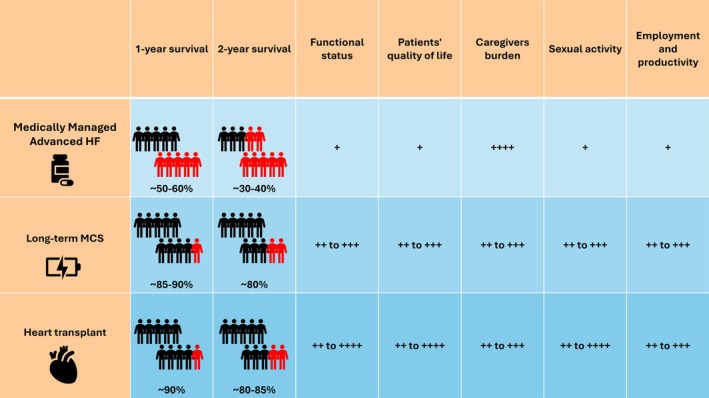
Comparative outcomes and quality of life metrics across advanced heart failure interventions. This table compares survival outcomes, functional status, quality of life for patients, caregivers' burden, sexual activity, and employment productivity for three advanced heart failure management strategies: advanced heart failure without mechanical support, long‐term mechanical circulatory support, and heart transplantation. Survival rates are presented as percentages, and median survival in years. Red figures: Proportion of patients who have died. Black figures: Proportion of patients who have survived. The ‘+’ symbols represent the degree of impact or improvement, categorized as follows: +: very low; ++: low; +++: moderate; ++++: high. HF, heart failure; MCS, mechanical circulatory support.

General data offer estimates on survival, QoL, complications, and rehospitalization rates (*Figure* *4*). However, these estimates are often derived from selected populations, excluding patients considered ineligible for treatment due to clinical or psychosocial factors. They may also fail to account for centre‐specific variations in resources and outcomes, and are frequently perceived by patients as too abstract or impersonal.

In contrast, personalized risk prediction tools are increasingly valuable for guiding discussions. They enable tailored comparisons based on individual profiles, helping patients and caregivers better anticipate outcomes and set realistic expectations. Such tools are particularly useful in ‘grey zone’ cases where the best course of action is uncertain. By reducing subjective bias influenced by social, emotional, or contextual factors, they offer a more objective foundation for shared clinical decisions.

Finally, clinicians must clarify that some symptoms, QoL impairments, and survival limitations may be due to comorbidities or organ dysfunctions rather than HF itself. These factors may only be partially improved by advanced therapies, and should be discussed transparently to help patients form realistic expectations and make truly informed choices.^61^

## Strengthening system‐level policies to enable equitable access to advanced heart failure therapies

Public health stakeholders play a pivotal role in strengthening system‐level policies to ensure equitable access to advanced HF therapies. However, multiple systemic barriers currently impede the realization of this goal. For instance, conventional health financing frameworks often fail to financially reward collaborative, network‐based care: there is frequently a lack of reimbursement for the team‐based, multidisciplinary activities required to manage advanced HF.^62^ Moreover, healthcare systems in many regions provide inadequate funding or incentives for integrated HF programs, and disparities in access to specialist care persist despite associated benefices.^63^, ^64^, ^65^ These financial and organizational gaps contribute to uneven availability of advanced therapies across different areas. In certain regions, patients have limited access to life‐saving interventions such as LVADs or HT due to insufficient local programs or funding constraints.^14^, ^66^ Compounding this, regional inequalities in telehealth infrastructure and support mean that remote monitoring and consultation services are not uniformly accessible, disproportionately affecting patients in rural or underserved communities.^66^

Addressing these challenges requires targeted policy solutions. Public health authorities should incentivize multidisciplinary care models by aligning reimbursement with high‐quality, coordinated care delivery (rather than volume‐driven services), thereby encouraging hospitals and networks to collaborate in advanced HF management. Providing reimbursement support for lengthy, complex advanced HF consultations and care coordination is also crucial, ensuring clinicians can devote the necessary time and resources to patient evaluation and education without financial disincentive. Furthermore, efforts must be made to ensure equity of access to advanced HF therapies regardless of geography. This could include expanding regional advanced HF centres or strengthening referral networks so that patients in historically under‐resourced areas can receive timely MCS implantation or transplant evaluation when indicated.^66^ Finally, bridging the digital divide is essential: investing in telehealth infrastructure and enacting supportive reimbursement policies for remote HF management will help guarantee that innovations like telemonitoring and virtual specialist consultations reach all patients. By implementing these system‐level strategies—incentivizing integrated care, bolstering financial support for advanced HF services, and ensuring new technologies and therapies are deployed equitably—public health stakeholders can markedly improve access to advanced HF treatments and move closer to true equity in care delivery.^46^, ^63^, ^66^

In conclusion, advanced HF requires early recognition and timely referral to specialized centres for therapies such as HT and MCS, which can improve both survival and QoL. However, many barriers remain, including fragmented care networks, limited access to severity assessment tools, lack of awareness of referral criteria, and underestimation of disease severity by patients.

Overcoming these challenges calls for coordinated efforts across all stakeholders to improve screening, clinician and patient education, shared‐care models, and health system support, ultimately ensuring more equitable access to advanced HF care.

**Conflict of interest**: G.B. has received personal fees from Abbott, AstraZeneca, Boehringer Ingelheim, Novartis and Novo Nordisk, outside of the submitted work. M.G.C.L. has received funds from the Carlos III Institute of Health of the Spanish Ministry of Science; personal fees from AstraZeneca, Boehringer Ingelheim, Astellas, Merck Sharp and Dohme, and Novo Nordisk, outside of the submitted work; and non‐financial support from Abbott and Pfizer, outside of the submitted work. C.D. declares lectures fees from Satelia, Abiomed and Abbott Medical; support for attending meetings from Abiomed and Abbott Medical; and consulting for AstraZeneca, Abbott Medical and Abiomed, outside of the submitted work. M.C. reports investigator‐initiated research grants to institution: Novartis, Abbott, Pfizer, clinical study contracts with institution: Novo Nordisk, CorVia, Advisory Role, speaker honoraria, travel grants: Abbott, Amgen, Amicus Therapeutics, AstraZeneca, Bayer, Boehringer Ingelheim, Bristol Meyers Squibb, GE Healthcare, Novartis, Novo Nordisk, Pfizer, Roche, Swixx, Takeda, Teva Pharmaceutical Industries, Viatris, outside of the submitted work. N.G. reports personal fees from AstraZeneca, Bayer, Boehringer, Novartis and Vifor, outside of the submitted work. F.G. has received consulting fees from Abbott, AstraZeneca, Bayer, Pfizer, Alnylam, Ionis, and Roche; and speaker fees from Novartis, outside of the submitted work. G.S. has received grants and personal fees from CSL Vifor, Boehringer Ingelheim, AstraZeneca, Servier, Novartis, Cytokinetics, Pharmacosmos, Medtronic, and Bayer; personal fees from Roche, Abbott, Edwards Lifesciences, Teva Pharmaceuticals, Menarini, INTAS Pharmaceuticals, GETZ Pharma, Laboratori Guidotti; and grants from Boston Scientific and Merck, all outside of the submitted work. L.W.v.L.'s employer UMC Utrecht has received grants and honoraria from Abbott, Medtronic, Vifor and Novartis. L.H. has received honorarium from Medtronic, AstraZeneca, and Alnylam, outside of the submitted work. A.Z. has received grant support from Xvivo and has received funding for participating in the speaker bureau for Paragonix, a company that manufactures organ preservation and transport devices, outside of the submitted work. M.M. has received consulting honoraria for participation in steering committees, advisory boards, or speeches from AstraZeneca, Bayer, Boehringer Ingelheim, Novo Nordisk and Roche Diagnostics, all outside of the submitted work. All other authors have nothing to disclose.
